# Supplementary material for: HIVprotI: an integrated web based platform for prediction and design of HIV proteins inhibitors
Source: J Cheminform. 2018 Mar 9;10:12. doi: 10.1186/s13321-018-0266-y (PMC5845081; doi:10.1186/s13321-018-0266-y)
Supplement: Supplementary file 2 — Additional file 2. Source code of HIVProtI web server. [file 13321_2018_266_MOESM2_ESM.zip › HIVprotI_Source-code/hivprotI/weka-3-6-6/doc/weka/classifiers/functions/SimpleLinearRegression.html]

SimpleLinearRegression


---


|  |  |  |
| --- | --- | --- |
| **Overview** \| **Package** \| **Class** \| **Tree** \| **Deprecated** \| **Index** \| **Help** \| **Weka's home** | |  |
| **PREV CLASS**   **NEXT CLASS** | **FRAMES**    **NO FRAMES**     **All Classes** |  |
| SUMMARY: NESTED \| FIELD \| CONSTR \| METHOD | DETAIL: FIELD \| CONSTR \| METHOD |  |


---


## weka.classifiers.functions Class SimpleLinearRegression

```
java.lang.Object
  weka.classifiers.Classifier
      weka.classifiers.functions.SimpleLinearRegression
```

**All Implemented Interfaces:**: java.io.Serializable, java.lang.Cloneable, CapabilitiesHandler, OptionHandler, RevisionHandler, WeightedInstancesHandler

---

``` public class SimpleLinearRegression extends Classifier implements WeightedInstancesHandler ```

Learns a simple linear regression model. Picks the attribute that results in the lowest squared error. Missing values are not allowed. Can only deal with numeric attributes.


Valid options are:

```
 -D
  If set, classifier is run in debug mode and
  may output additional info to the console
```

**Version:**
:   $Revision: 5523 $

**Author:**
:   Eibe Frank (eibe@cs.waikato.ac.nz)

**See Also:**: Serialized Form

---

**Constructor Summary**

`SimpleLinearRegression()`


| **Method Summary** | |
| --- | --- |
| `void` | `buildClassifier(Instances insts)`             Builds a simple linear regression model given the supplied training data. |
| `double` | `classifyInstance(Instance inst)`             Generate a prediction for the supplied instance. |
| `boolean` | `foundUsefulAttribute()`             Returns true if a usable attribute was found. |
| `int` | `getAttributeIndex()`             Returns the index of the attribute used in the regression. |
| `Capabilities` | `getCapabilities()`             Returns default capabilities of the classifier. |
| `double` | `getIntercept()`             Returns the intercept of the function. |
| `java.lang.String` | `getRevision()`             Returns the revision string. |
| `double` | `getSlope()`             Returns the slope of the function. |
| `java.lang.String` | `globalInfo()`             Returns a string describing this classifier |
| `static void` | `main(java.lang.String[] argv)`             Main method for testing this class |
| `void` | `setSuppressErrorMessage(boolean s)`             Turn off the error message that is reported when no useful attribute is found. |
| `java.lang.String` | `toString()`             Returns a description of this classifier as a string |

**Methods inherited from class weka.classifiers.Classifier**

`debugTipText, distributionForInstance, forName, getDebug, getOptions, listOptions, makeCopies, makeCopy, setDebug, setOptions`

**Methods inherited from class java.lang.Object**

`equals, getClass, hashCode, notify, notifyAll, wait, wait, wait`

**Constructor Detail**

### SimpleLinearRegression

```
public SimpleLinearRegression()
```


**Method Detail**

### globalInfo

```
public java.lang.String globalInfo()
```

:   Returns a string describing this classifier

    :   **Returns:**: a description of the classifier suitable for displaying in the explorer/experimenter gui

---


### classifyInstance

```
public double classifyInstance(Instance inst)
                        throws java.lang.Exception
```

:   Generate a prediction for the supplied instance.

    :   **Overrides:**: `classifyInstance` in class `Classifier`
    :   **Parameters:**: `inst` - the instance to predict. **Returns:**: the prediction **Throws:**: `java.lang.Exception` - if an error occurs

---


### getCapabilities

```
public Capabilities getCapabilities()
```

:   Returns default capabilities of the classifier.

    :   **Specified by:**: `getCapabilities` in interface `CapabilitiesHandler` **Overrides:**: `getCapabilities` in class `Classifier`
    :   **Returns:**: the capabilities of this classifier **See Also:**: `Capabilities`

---


### buildClassifier

```
public void buildClassifier(Instances insts)
                     throws java.lang.Exception
```

:   Builds a simple linear regression model given the supplied training data.

    :   **Specified by:**: `buildClassifier` in class `Classifier`
    :   **Parameters:**: `insts` - the training data. **Throws:**: `java.lang.Exception` - if an error occurs

---


### foundUsefulAttribute

```
public boolean foundUsefulAttribute()
```

:   Returns true if a usable attribute was found.

    :   **Returns:**: true if a usable attribute was found.

---


### getAttributeIndex

```
public int getAttributeIndex()
```

:   Returns the index of the attribute used in the regression.

    :   **Returns:**: the index of the attribute.

---


### getSlope

```
public double getSlope()
```

:   Returns the slope of the function.

    :   **Returns:**: the slope.

---


### getIntercept

```
public double getIntercept()
```

:   Returns the intercept of the function.

    :   **Returns:**: the intercept.

---


### setSuppressErrorMessage

```
public void setSuppressErrorMessage(boolean s)
```

:   Turn off the error message that is reported when no useful attribute is found.

    :   **Parameters:**: `s` - if set to true turns off the error message

---


### toString

```
public java.lang.String toString()
```

:   Returns a description of this classifier as a string

    :   **Overrides:**: `toString` in class `java.lang.Object`
    :   **Returns:**: a description of the classifier.

---


### getRevision

```
public java.lang.String getRevision()
```

:   Returns the revision string.

    :   **Specified by:**: `getRevision` in interface `RevisionHandler` **Overrides:**: `getRevision` in class `Classifier`
    :   **Returns:**: the revision

---


### main

```
public static void main(java.lang.String[] argv)
```

:   Main method for testing this class

    :   **Parameters:**: `argv` - options


---


|  |  |  |
| --- | --- | --- |
| **Overview** \| **Package** \| **Class** \| **Tree** \| **Deprecated** \| **Index** \| **Help** \| **Weka's home** | |  |
| **PREV CLASS**   **NEXT CLASS** | **FRAMES**    **NO FRAMES**     **All Classes** |  |
| SUMMARY: NESTED \| FIELD \| CONSTR \| METHOD | DETAIL: FIELD \| CONSTR \| METHOD |  |


---
